# Neuroinflammation as the Proximate Cause of Signature Pathogenic Pattern Progression in Amyotrophic Lateral Sclerosis, Aids, and Multiple Sclerosis

**DOI:** 10.1155/2012/169270

**Published:** 2012-12-04

**Authors:** Lawrence M. Agius

**Affiliations:** Department of Pathology, Mater Dei Hospital, University of Malta Medical School, Msida MSD 2090, Malta

## Abstract

The realization of injury to large motor neurons is embedded within contextual reference to the parallel pathways of apoptosis and necrosis of system-patterned evolution. A widespread loss of cell components occurs intracellularly and involves a reactive participation to a neuroinflammation that potentially is immunologically definable. In such terms, sporadic and hereditary forms of amyotrophic sclerosis are paralleled by the components of a reactive nature that involve the aggregation of proteins and conformational misfolding on the one hand and a powerful oxidative degradation that overwhelms the proteasome clearance mechanisms. In such terms, global participation is only one aspect of a disorder realization that induces the development of the defining systems of modulation and of injury that involves the systems of consequence as demonstrated by the overwhelming immaturity of the molecular variants of mutated superoxide dismutase. It is further to such processes of neuroinflammatory consequence that the immune system is integral to the reactive involvement of neurons as patterns of disease recognition and as the system biology of prevalent voluntarily motor character. It is highly significant to recognize various inflammatory states in the nervous system as prototype variability in phenotype expression and as incremental progression in pathogenesis. In fact a determining definition of amyotrophic lateral sclerosis is an incremental phenotype modulation within the pathways of the consequential loss and depletion of motor cell components in the first instance. Neuroinflammation proves a pattern of the contextual spread of such pathogenic progression in the realization of end-stage injury states involving neurons and neuronal networks.

## 1. Introduction

Amyotrophic lateral sclerosis (ALS) is manifested by an array of the large neuronal cell loss of motor type as further constituted by a toxic gain of the function of superoxide dismutase in a small percentage of the patients. The evolutionary course of the disease is further constituted by a series of progressive changes that affect clinically both sporadic and hereditary forms of motor neuronal loss [1]. In such manner, composite manifestations attest to a widespread semblance to neuronal trophic factor deficiency that is superimposed by a constitutive dying back phenomenon of axonal degeneration in the virtual absence of sensory nerve involvement.

## 2. Disease Pattern

Disease pattern is recognizable in terms of ongoing changes in neuronal cells that comprises a noncell autonomous involvement implicating glial cells ranging from astrocytic glutamate production and microglial reactivity. Nuclear factor-kappaB downregulation in neuronal nuclei in ALS might promote the loss of neuroprotection or else be associated with nuclear loss [2].

The developmental consequences of injury in this disorder are further evidenced by the appearance of defects in axonal transport and in the phosphorylation of light and heavy forms of neurofilament, by the appearance of aggregation or inclusion bodies and also by such structures as hyaline bodies consisting of mutant superoxide dismutase, skein inclusions, Bunina bodies, and at times discrepancy in the evolutionary course when correlated with specific missense mutations of superoxide dismutase gene.

The overall dynamics of this heterogeneous group of disorders complicate a derivational body of consequences that results, in the overwhelming majority of cases, in respiratory failure after some 2-3 years of disease course. The skeletal muscle atrophy is a realization of the central nervous system involvement that evolves as an apparent consequence to lesions in motor supplying neuronal axons and to abnormalities in eventual neuromuscular junctions supplying these muscles. Disease-driven changes in ATP-binding cassette drug efflux transporters in the CNS interfere with effective ALS pharmacotherapies [3].

A network basis for the disease is recognizable in terms of evolutionary dynamics in the face of injury to a systemic series of structural components throughout the entire complex of motor neuronal supply to skeletal muscle. In such manner, patterns of involvement are realized dimensions of network participation in the delineation of progressive kinetics in skeletal muscle atrophy.

The disease recognition patterns of involvement are reflected in the systemic response of neurons to endoplasmic reticulum stress and cytosol saturation by mutant superoxide dismutase in some 20% of the hereditary cases of amyotrophic lateral sclerosis. In such manner, oxidative stress is a recognized component pathway with indices referable to such stress response to a primary injury to neurons. It is in such capacity of attempted containment of the cellular stress that the progression of this disorder proves the true dimensional dynamics of pathogenesis in neuronal cell loss. Oligoclonal bands in the CSF of patients with ALS may be associated with gene mutations [4]. Further participation of other anti-oxidants also feature in a series of response pathways that correlate with the production of such molecules as free oxygen radicals and peroxynitrite in a mode of systemic involvement involving cell signalling pathways and deranged intracellular transport mechanics. A central issue is the globality of posttranslational modifications of neurofilaments and an oxidative burst of induced neuronal apoptosis and necrosis. The overall parameters of the induced performance and further execution of oxidative stress and injury are integral components of derived consequence in the injury response of neurons to intracellular stress.

Microglia become transformed and neurotoxic in end-stage disease ALS [5].

Aggregation of immature forms of superoxide dismutase and the mechanics of cytosol-nuclear abnormalities of the exchange and transport of antioxidants also are referable to the dimensions of a recognition pattern signature of disease involvement that attests to the overall dynamics of neuronal cell death pathways [6].

Lipid and DNA oxidation correlate with the systems of the aggregation of inclusion bodies as further attested by the oxidation of the superoxide dismutase itself. It is further to ongoing developmental outlines of disease reappraisal that amyotrophic lateral sclerosis is indeed a response pattern of consequence within the contextual reproducible pathways of ongoing aberrant intracellular transport mechanics. Neuroinflammation proves a response to neuronal dysfunction and death and response manipulation might alter disease progression [7]. An essential aspect of participating influence in the ongoing progression of amyotrophic lateral sclerosis is the potentially inflammatory array of constitutive factors in pathogenesis that are allied to the system forms of involvement as in central nervous system involvement in AIDS patients and in multiple sclerosis victims.

## 3. Dynamics of Neuroinflammation in General

The concept of the renewal of the immune response arises in terms of a recurring system of parallel pathways that may especially target the central nervous system. 

It is with regard to an extensive repertoire of conditioning and reconditioning manoeuvres that the overall dimensionality of targeting provokes a reappraisal of current processes that prove dominant in further injuring the brain in patients with acquired immunodeficiency syndrome [8]. A critical array of parallel and superimposed parameters proves decisive in determining a series of abnormal system responses on the part of the central nervous system itself. The increased chemotactic signals in HIV infection of the brain promote a marked transmigration into the CNS [9]. In such modes of participation, the integral involvement of both the innate and adaptive systems of immune response proves paramount determinant in providing an excessive or deficient overall response that operatively compromises the systems of the conditioning multifocality of tissue [10]. It is within the conceptual integrity of the multiple different cell and tissue components of the brain that the realization of injury is potentially protected by a diversity of immune response and is also further provoked by the dimensions of inflammation that exceed dynamic targeting capability. Interferon-gamma signalling and proinflammatory effect injure oligodendrocytes in multiple sclerosis [11]. Metabotropic glutamate receptors modulate glial cell proliferation, glutamate uptake, neurotrophic support, and neuroinflammation [12]. 

It is further to be realized that the systems of the involvement of inflammatory reactivity are borne out by a system organ that is participant in provoking systemic by-products as defined by multiple organs such as the reactive spleen and lymph nodes.

Such panorama of participating components in AIDS encephalitis is a truly derived phenomenon and also a real originator of further aberrant responsiveness as evidenced by a wide diversity of possible opportunistic infections seen clinically in patients with AIDS.

It is within the developmentally aberrant severity of the inflammatory response within the central nervous system that the realization of tissue injury proves a progressive phenomenon of self-sustaining participation in injuring further the native neural tissues [13]. The significance of the realization of a focus of encephalitis or cerebritis is the concurrent process of integrative evolution in the further establishment of the parameters of incremental progression even in a generic sense. Monocyte turnover and monocyte/macrophage activation are implicated in progression in HIV encephalitis [14]. This is also evidenced in patients suffering from recurrent attacks of multiple sclerosis within the scope of developmental outcome in system involvement as evidenced by oligodendrocyte depletion and the loss of myelin sheaths [15].

B cells and antibodies are implicated in at least a subset of patients with multiple sclerosis and are related to the production of oligoclonal bands. The CNS also locally produces antibodies [16].

The extreme variability of pathologic events both in patients with AIDS encephalitis and also in patients with different biologic substrate in evolving multiple sclerosis proves a serial representation of multimodal realizations that provoke an immune participation that results in a further augment in the inflammatory response. Lipid metabolism and vascular pathology are both implicated in multiple sclerosis [17].

In such manner, the provocation of a targeting inflammatory process is determined and also determines in its turn an alternating but self-sustaining realization of tissue injury that spans the dimensional conditions in AIDS encephalitis or multiple sclerosis. It is further to a serial reconstruction of various forms of injury to neural tissues that the by-product of significant injury is the main criteria in the evidential proposition of inflammatory and immune system induction to further tissue injury [18]. Oxidative stress may target mitochondria and induce both neurodegeneration and demyelination [19]. 

The distinctive cell subpopulations of the involvement of the central nervous system are real component of a confined series of pathways of an organ such as the brain that proves not immune privileged [20]. Thus, paradoxically, the participant organ system is a realization of the ongoing cooperative participation of the immune and inflammatory responses. A large body of evidence implicates autoimmunity in multiple sclerosis [21]. It is further to system integrity that the overall effects of AIDS encephalitis and the recurrent relapses of multiple sclerosis both prove a highly persistent form of active tissue injury in terms specifically of the tissue component identities of the central nervous system [22]. The realization of an injury to neural tissues is a significant dimension of the reproducible persistence of the disease process. Mechanisms of the pleiotropic effects of sodium channels involve interactions between glia and neurons via cytokines, growth factors, and neurotransmitters at synapses and axons [23]. The provocative further promotion of injury to tissues is represented or constituted by such systems as astrocytic and microglial response and as plaque enlargement and demyelination.

The constitutive evidence for a further increment in disease activity is borne out by developmental innate immune response and as further signified by the injury to multiple tissue components [24].

The multifocality of injury to the CNS is an integration of evidential pathways of the reconstruction of the injurious events themselves in various modes of the further promotion of such pathways as directed microglial response and as participating vascularity and as also gliosis and subsequent aberrant immune- and inflammatory-mediated responses [25]. HIV treatment failure allows the compartmentalized viral replication and development of resistance [26].

Further to the significant emergence of tissue injury, the parameters of dimensional involvement in CNS inflammation are dramatically reconstituted in terms of the realization of new, targeted responses to other foci of directed involvement. It is significant to consider the overall dimensions of realization in terms of the participation of the overall self-augmenting or positive feedback loops in CNS inflammatory states [27].

Dual participation of the immune and the inflammatory pathways proves integrative, especially with an increasing severity of these responses towards further foci of neural injury. The dimensions of further cooperative participation are mutually self-identifying motives in the significant emergence to necrotic foci of neural tissue. 

Microglia express various Pattern Recognition Receptors to identify viral signatures called Pathogen Associated Molecular Patterns to which microglia respond by producing inflammatory mediators [28].

In terms of such ongoing pathway culmination, the persistence of an integrative response is paradoxically self-generating as realized also by the intermittent relapses in multiple sclerosis patients or in the recurrent attacks of opportunistic infection in AIDS patients.

## 4. Neuroinflammation in Motor Neuronal Loss

Neuroinflammation is a potentially constitutive mode of pathogenesis in various neurodegenerative disorders such as amyotrophic lateral sclerosis that evolves as an apparent primary neuronal cell loss within the additional contexts of the parallel evolution of apoptosis and necrosis of the neurons [29].

In such manner, a beneficial incremental change attests to the possible limitation of injury that is partly contributed to by glial cells such as astrocytes and by microglial reactivity.

The corresponding pathologic spread in amyotrophic lateral sclerosis of a putative agent is analogous to dynamics of involvement in prion disease in terms of aggregation that corresponds to mechanics of neuronal cell loss in these disorders. 

The innate immune response is a recognized component pattern of involvement in a disease process such as neurodegeneration whereby also inclusion bodies are the consequences of attested intracellular stress mechanics.

Disease recognition patterns as network involvement primarily indicate system participation in pathogenic progression and also as incremental definition of inflammatory pathways. In such terms, the distribution of motor neuron loss indicates a predilection for neurons in reference to such indices as the large size of the cells and as further spread involvement within the neuronal motor systems [30].

Toxic gain of function of mutant superoxide dismutase indicates a realization that is deferentially distributed due to modes of action independent of enzymatic function. In such manner, the promotional realization of evidential pathways includes derived neuronal lesions that arise as neuroinflammatory foci and as distributional realization for the further spread of the neuronal cell loss. 

Composite idealization is dimensionally ensured as a significant pattern formulation in disease signature definition. It is in view of comparable indices as parameters of evolution that disease progression permits and also enhances susceptibility to stress-induced injury to individual neurons within motor system pathways.

Blood brain barrier breakdown leads to a neuroinflammation and oxidative stress [31].

Distributional markers as models of injury indicate a proximate series of changes that account in turn for the oxidative series of modulated lesions within the patterns of the evolutionary progression of the disease. 

Hereditary motor neuronal lesions prove a susceptibility series of a heightened nature and are formulated by the network reactivity of neuronal subsets.

System biology is a source of potential realization in amyotrophic lateral sclerosis that persistently constitutes the determined involvement of systems of the progression of a disease process that is rapid and incrementally realized as a loss of large motor neurons. It is further to such considerations that the outline parameters of overall index involvement include the definition of patterns of disease definition within the distributional reality of the motor neuronal system as a whole.

The overactivity parameters of oxidative stress somehow include a toxic gain of function that involves systems of repair or compensation, on the one hand, and also serial modulation of recoverability as system dimensions of the disease entity. 

It is in terms of such evolutionary course that the further outline of lesion characterization permits the distribution of significant lesions beyond simple oxidative stress.

The sporadic form of amyotrophic lateral sclerosis includes oxidative stress in its own right but as definable beyond mutations of the superoxide dismutase enzyme. It is with regard to further neuroinflammatory injury that the immune system appears as a common referential series of pathways in its own right that defines the nature of characterized neuronal cell loss.

Neuroinflammation pathogenically links such diverse conditions such as amyotrophic lateral sclerosis, AIDS encephalitis, and multiple sclerosis with the marked activation of astrocytes and microglia and the production of proinflammatory agonists. Upregulation of endothelial adhesion molecules and downgrading of tight junction proteins facilitate in particular CNS the ingress of T lymphocytes [32].

In such manner, parametric indices allow for the emergence of overall dimensions as important determinants in the characterization of the progressive course of a disorder that is primarily depletive but also reactive. Neuroinflammation, hence, is constitutionally a superimposed series of gains in toxicity that overcome recoverability parameters on the part of individual neurons as integrated signature networks of pattern recognition.

Significant overall dimensions of inclusion allow for the distribution of lesions within the intracellular compartment in modes of aggregation and precipitation as evidenced by forms of mutated superoxide dismutase that lack in particular the Zn ion or of the disulfide bonds. 

It is further to a compromising realization of immature metabolic phenotype that mitochondrial damage precipitates apoptotic cell death and also other metabolic phenotypes of the realized destabilization of the proteasomal system in misfolded protein clearance. 

With reference to misfolded moieties of protein aggregation it is significant to recognize the reactive constitution of the motor system disorders within the context of further progression of the neuronal cell loss.

## 5. Concluding Remarks

Various aspects of the biology of neuronal cell loss permit the global evolution of injury that complicates patterns of potential recoverability in the face of evolutionary system participation. Allowance for participation is significant in terms of further modulation of the lesion that accommodates indices of determining pathogenic influence. Neuroinflammation is a powerfully effective series of revision pathways that incorporate the subsequent realization of the evolutionary patterns of an incremental nature. 

In terms of serial conformation and as evidential system patterns, the biology of amyotrophic lateral sclerosis consists of an inflammatory reactivity in the face of the serial reconstitution of inflammatory and immune pathways that incrementally challenge the neuronal viability issues. 

Such processes implicate the significant modification of phenotype as evidential systems of systemic requirement. It is with regard to system pathways that the conclusive phenomenon of neuronal cell death indicates the deliberate termination of system determination.

In overall terms, the required pathogenic course dynamics in neuronal cell loss participate as systems of over-riding reactivity. Significant signature recognition patterns of pathogenic progression are potentially implicated in amyotrophic lateral sclerosis. 

Such incremental indices would indicate the overwhelming systemic central nervous system involvement as predominant neuroinflammatory indices of activity and reactivity.
